# Early evidence of efficacy for orally administered SPM-enriched marine lipid fraction on quality of life and pain in a sample of adults with chronic pain

**DOI:** 10.1186/s12967-020-02569-5

**Published:** 2020-10-21

**Authors:** Nini Callan, Doug Hanes, Ryan Bradley

**Affiliations:** 1 National University of Natural Medicine, Helfgott Research Institute, 2220 SW 1st Ave, Portland, OR 97201 USA; 2 Herbert Wertheim School of Public Health, University of California, San Diego, La Jolla, CA USA

**Keywords:** Chronic pain, Pain, Spms, Resolvins, Specialized pro-resolving mediators, Fish oil, Quality of life, Mood

## Abstract

**Background:**

Marine lipids contain omega-3 fatty acids that can be metabolized into anti-inflammatory and pro-resolving mediators—namely 17-HDHA and 18-HEPE—which can serve as modulators of the pain experience. The purpose of this study was to determine the impact of 4 weeks of oral supplementation with a fractionated marine lipid concentration, standardized to 17-HDHA and 18-HEPE, on health-related quality of life and inflammation in adults with chronic pain.

**Methods:**

This study was a prospective, non-randomized, open-label clinical trial. Forty-four adults with ≥ moderate pain intensity for at least 3 months were recruited. The primary outcome was change in health-related quality of life (QOL) using the Patient Reported Outcomes Measurement Information System-43 Profile (PROMIS-43) and the American Chronic Pain Association (ACPA) QOL scale. Exploratory outcomes assessed safety and tolerability, changes in anxiety and depression, levels of pain intensity and interference, patient satisfaction, and impression of change. Changes in blood biomarkers of inflammation (hs-CRP and ESR) were also explored.

**Results:**

Outcome measures were collected at Baseline, Week 2, and Week 4 (primary endpoint). At Week 4, PROMIS-43 QOL subdomains changed with significance from baseline (*p* < 0.05), with borderline changes in the ACPA Quality of Life scale (*p* < 0.052). Exploratory analyses revealed significant changes (*p* < 0.05) in all measures of pain intensity, pain interference, depression, and anxiety. There were no statistically significant changes in either hs-CRP or ESR, which stayed within normal limits.

**Conclusion:**

We conclude that oral supplementation with a fractionated marine lipid concentration standardized to 17-HDHA and 18-HEPE may improve quality of life, reduce pain intensity and interference, and improve mood within 4 weeks in adults with chronic pain. The consistency and magnitude of these results support the need for placebo-controlled clinical trials of marine lipid concentrations standardized to 17-HDHA and 18-HEPE.

*Trial registration* ClinicalTrials.gov: Influence of an Omega-3 SPM Supplement on Quality of Life, NCT02683850. Registered 17 February 2016—retrospectively registered, https://clinicaltrials.gov/ct2/show/NCT02683850.

## Background

According to the National Institutes of Health, chronic pain affects an estimated 100 million Americans—or one-third of the U.S. population—and societal costs are estimated to be up to $630 billion per year [1]. Approximately 25 million of these people experience moderate to severe *chronic* pain that limits activities, diminishes quality of life, and for which effective and safe treatments are limited.

Alternatives for the management of chronic pain are needed due to the high side effect profiles, high incidence of developing tolerance, and high potential for addiction in the most common treatments currently used [2]. Non-steroidal anti-inflammatory drugs (NSAIDs) alone are responsible for an estimated 100,000 hospitalizations and 15,000 deaths annually, and increases in opioid prescriptions for chronic pain have been accompanied by increases in overdoses, abuse, and early death, including death from accidental overdose [3, 4]. In addition, once pain becomes chronic, research has shown NSAIDS are no longer effective, and many drugs show limited to no effectiveness [5, 6]. Furthermore, none of these prescriptions or over-the-counter medications are recommended for long-term use, nor do they resolve the chronic inflammation often associated with chronic pain.

Marine lipids (i.e. fish oil) are a well-known source of the long chain omega-3 fatty acids eicosapentaenoic acid (EPA) and docosahexaenoic acid (DHA), and have compelling potential for further study in the treatment of chronic pain. EPA and DHA can be metabolized in the body into potent anti-inflammatory and pro-resolving mediators (18-HEPE and 17-HDHA, respectively), which are integral parts of a fatty acid metabolite class known as specialized pro-resolving mediators (SPMs)—SPMs have been shown to have analgesic effects in several models of inflammatory and neuropathic pain, as well as the ability to modulate the detection and induction of pain [7–13]. The mechanism for such effects may be SPMs ability to alter the inflammatory response through direct and immunological paths, including counter-regulating the actions of pro-inflammatory mediators, enhancing leukocyte phagocytosis and efferocytosis, increasing the production of anti-inflammatory mediators, increasing the killing and clearance of microbes, modulating TRP channels, and enhancing tissue regeneration [12, 14, 15]. In addition to direct analgesic effects, by modifying the inflammatory response and thereby encouraging long-term injury resolution, the transition from pain to chronic pain may be slowed or halted as well.

SPMs have been standardized within a fractionated marine lipid concentration and are available for oral supplementation. A crossover study done in a healthy adult population supplemented with an emulsion format of the fractionated marine lipid concentration used in the present study; 24 h results revealed increases in circulating SPM concentrations, a reprogramming of immune cells towards enhanced phagocytosis, and a moderated pro-inflammatory response [16]. Such results highlight the bioavailability and functional effects of supplementation. No clinical research has been published on the effects this fractionated marine lipid concentration might exert on a chronic pain population, and thus, this trial sought to collect preliminary data on the effects of supplementation on quality of life, pain, mood, and inflammation in adults with a history of chronic pain.

## Methods

### Design and sampling

The design of this research was a single-arm, open-label clinical trial. The protocol and all study materials were reviewed and approved by the IRB of the National University of Natural Medicine (NUNM; MetaG SPM IRB, #091515-B) and registered on ClinicalTrials.gov as *Influence of an Omega-3 SPM Supplement on Quality of Life* (NCT02683850). The trial aimed to assess the impact of SPM Active**™** softgel supplementation on quality of life in adults with moderate to severe chronic pain symptoms (as measured by the Patient Reported Outcomes Measurement Information System (PROMIS)-43 Profile—Pain Intensity subdomain).

Participants were recruited from the greater Portland, Oregon area using community-based flyers and advertisements. Information about the study was also available through the NUNM website. In addition, a network of community physicians was established for additional recruitment. Potential participants were screened over the telephone and referred to one of five clinical sites to determine eligibility.

Eligible candidates included adults 20–70 years of age, with a Body Mass Index of 19–40 kg/m^2^, that had no other significant medical problems, were able to maintain stable intake of therapeutic agents for at least 30 days, and were able to refrain from adding any therapeutic agents for the duration of the study. In addition, only participants suffering from moderate to severe chronic pain (i.e. an average pain score of greater than or equal to a 4 on the PROMIS-43 Profile—Pain Intensity subdomain) for at least 3 months were included in the study. Exclusion criteria were chosen to minimize the possibility of confounding the detection of changes in pain or inflammation, such as recent initiation of, or changes to, pain medications or other pain reduction therapies. In addition, women who were lactating, pregnant, or planning pregnancy at the time of screening or would be within the 6 months subsequent to screening, were excluded from study participation.

### Intervention, packaging, and labeling

Study participants received 2 bottles of the intervention: SPM Active™ softgels (Metagenics, Inc., Gig Harbor, WA). The SPM Active™ softgel is a dietary supplement Generally Recognized As Safe (GRAS), commercialized by Metagenics Inc. The SPM Active™ softgels used in this study were manufactured by Solutex (https://www.solutex.es/; Parque Empresarial Omega Edificio Gamma Avenida de Barajas 24, 3ª 28109 Madrid, Spain). These softgels met or exceeded all quality control requirements, as well as all softgel production requirements for Good Manufacturing Practices (cGMP).

Each SPM Active™ softgel contained 250 mg of a marine lipid fraction (Lipinova®), standardized to 17-HDHA and 18-HEPE (Solutex, Spain) with demonstrated pro-resolving activity covered by the patent family PCT/US2013/040313.

For the purposes of this study, the softgels were packaged in unlabeled bottles with approximately 60 softgels per bottle. A product label was provided by the Helfgott Research Institute, which included instructions on use, as well as contact information for any questions that arose.

Participants were provided with written instructions at their Baseline visit and were instructed to take 3 softgels in the morning and 3 softgels in the evening. Participants were also provided with a study supplement log to record their daily intake of the softgels and record any questions or concerns that emerged. Participants returned the study supplement log and any unused study supplement softgels at the Week 2 and Week 4 study visits.

SPM Active™ dose titration occurred during the Week 2 study visit, based on the pain ratings obtained via REDCap reported within the 2 days prior to the visit*.* Participants who reported PROMIS-43-measured ‘pain intensity’ levels that had decreased by 2 points or more after 2 weeks had their dose decreased to 2 softgels in the morning and 2 softgels in the evening for weeks 3 and 4 of the study (N = 16). Participants who reported PROMIS-43-measured ‘pain intensity’ levels that had not changed, had only decreased by one point, or had increased, increased their dose to 4 softgels in the morning and 4 softgels in the evening for weeks 3 and 4 of the study (N = 28). The unused supplement bottle from the first 2 weeks was relabeled with the appropriate dosing and returned to participants for the last 2 weeks of the trial.

### Study visits

The study participant visits were grouped into 2 categories: screening visit and study visits. Study visit one (Baseline) occurred directly after the screening visit. Clinical re-evaluations occurred every 2 weeks, as Week 2 and Week 4 study visits.

At the screening visit, medications and supplements were reviewed, as was participant health history. Eligibility was determined by administration of a standardized ninety-one point Adverse Event Monitoring form (participants were excluded if any item was determined to be Grade 3, ‘severe or medically significant but not immediately life-threatening’, or higher); the PROMIS-43 Profile–Pain Intensity subdomain; and BMI. After an informed consent consultation, eligible participants were enrolled in the study.

The Baseline study visit and subsequent study visits included administration of the Adverse Event Monitoring form, PROMIS-43 Profile, American Chronic Pain Association’s (ACPA) Quality of Life Scale, Patient Health Questionnaire (PHQ-9), Generalized Anxiety Disorder scale (GAD-7), and Brief Pain Inventory long form (BPI). In order to reduce provider interference on participant responses, the administration of all research instruments was separated from clinical care and provider interaction through the use of a centralized REDCap-based survey sent to and completed by participants on their own (but according to the study timeline). A blood sample was taken at each visit to be analyzed for the biomarkers high-sensitivity C-reactive protein (hs-CRP) and erythrocyte sedimentation rate (ESR).

The Patient Global Impression of Change (PGIC) and the Patient Global Satisfaction Scale (PGSS) were administered at the Week 4 study visit only.

### Outcome measures

#### Primary outcome measure

The primary outcome of the trial was health-related quality of life, measured by the PROMIS-43 instrument (primary measure) and the ACPA’s Quality of Life Scale (secondary measure).

##### PROMIS–43 Profile subdomains

The PROMIS-43 Profile consists of seven domains (Ability to Participate in Social Roles and Activities, Anxiety, Depression, Fatigue, Pain Interference, Physical Function, and Sleep Disturbance), with six questions per domain rated on a 5-point rating scale. Additionally, there is a 1-question Pain Intensity domain rated on an 11-point scale. The domains are assessed “over the past 7 days” except for the Physical Function domain, which has no specified time frame. A raw score is created from each subscale (except Pain Intensity) that makes up the Profile. Raw scores are translated into T-scores, which are reported as the final score for each participant. The PROMIS-43 Profile provides a standardized, reliable, and valid measure of Pain Interference, Pain Intensity, Physical Function, Fatigue, Sleep Disturbance, and Ability to Participate in Social Roles and Activities. The Dutch-Flemish PROMIS Pain Behavior item bank was found to have good cross-cultural validity, reliability and construct validity [17].

The subdomains used to assess quality of life as the primary outcome included Ability to Participate in Social Roles and Activities (shortened for purposes of this study to Social Function), Fatigue, and Sleep Disturbance. The subdomains of Anxiety, Depression, Pain Interference, and Physical Function, as well as Pain Intensity, were used in conjunction with additional standardized tools as exploratory outcomes, as described below.

##### American Chronic Pain Association’s quality of life scale

The ACPA’s Quality of Life Scale is a single item measure of function for people with chronic pain. Quality of Life is rated using an 11-point scale ranging from “Non-Functioning” to “Normal Quality of Life”. The ACPA Quality of Life scale was developed specifically as a measure of functioning for people with chronic pain. It has been used by thousands of medical professionals across the globe for many years and is used extensively by the US Department of Veterans Affairs. Wayne State University College of Nursing is currently using the scale in research of the maintenance and improvement of functional states in patients with chronic pain.

#### Exploratory outcome measures

Exploratory outcomes included: changes in depression and anxiety; pain relief; pain intensity; pain interference; physical function; patient satisfaction; patient impression of change in their condition; changes in inflammatory serum biomarkers; and adverse events. Changes in depression were measured by the PHQ-9 and the PROMIS-43 Profile–Depression subdomain, while changes in anxiety were measured by the GAD-7 scale and the PROMIS-43 Profile–Anxiety subdomain. Items in the BPI determined pain relief and pain quality. Pain intensity and pain interference were determined by the PROMIS-43 Profile subdomains of the same names, as well as several BPI items, as outlined below. Patient satisfaction and impression of change were determined using the PGSS and the PGIC. The biomarkers hs-CRP and ESR were used to assess changes in inflammation. A comprehensive case report form was used to determine changes in pain medication use. Adverse events were closely monitored and systematically collected. These tools are described in detail below.

##### Patient Health Questionnaire (PHQ-9)

The PHQ-9 is a self-administered depression scale based on the mood module from the PRIME-MD diagnostic instrument for common mental disorders. The PHQ-9 scores each of the nine DSM-IV criteria as “0” (not at all) to “3” (nearly every day), rated for the last 2 weeks—this provides a depression severity score based on a 0–21 continuous scale. The PHQ-9 is a validated instrument for detecting depression and monitoring its severity, and higher scores are associated with increasing levels of depression severity [18, 19]. The PHQ-9 final score is rated from No Depression to Severe Depression.

##### Generalized Anxiety Disorder scale (GAD-7)

The GAD-7 is a 7-item self-report instrument to assess generalized anxiety disorder in primary care patients. Items are rated for the last 2 weeks, using a 4-point rating scale from “1” (not at all) to “5” (nearly every day). A score of 10 or greater on the GAD-7 represents a cut point for identifying cases of generalized anxiety disorder, while cut points of 5, 10, and 15 might be interpreted as representing mild, moderate, and severe levels of anxiety on the GAD-7. There is an overall relationship between GAD-7 severity levels and disability scores, with higher mean disability values related to higher severity levels [20].

##### Brief Pain Inventory (BPI), long form

The BPI is a 32-item self-report questionnaire that examines pain severity/intensity and impairment caused by pain on emotional and physical functioning. The instrument consists of a series of 11-point numeric rating scales. 4 items measure pain intensity (pain now, average pain, worst pain, and least pain) using “0” (no pain) to “10” (pain as bad as you can imagine) as anchors. These scores are individually given as measures of pain. Seven items measure the level of interference with function caused by pain during the past week (general activity, mood, walking ability, normal work, relations with other persons, sleep, and enjoyment of life) with anchors of “0” (does not interfere) to “10” (completely interferes). A composite mean score of the seven items is given as a measure of pain interference.

The BPI also asks the patient to rate the quality of their pain (e.g. aching, throbbing, shooting, stabbing, etc.) and to rate the relief they feel from the current pain treatment. The BPI pain scale has been widely used and found to provide a reliable and valid measure of pain, pain interference, and improvements in pain over time across cultures and languages, and for purposes of this study, in chronic nonmalignant pain populations [21, 22].

##### PROMIS–43 Profile subdomains

This instrument is described in detail above (*Primary Outcome Measure* section). Pain Intensity and the subdomains of Anxiety, Depression, Physical Function, and Pain Interference were used as exploratory outcome measures. The Anxiety and Depression subdomains were used in conjunction with the GAD-7 and PHQ-9 to measure anxiety and depression. Pain Intensity and Pain Interference were used in conjunction with the BPI items to measure pain intensity and interference; Physical Function was used as an independent marker to determine changes in physical function.

##### Patient Global Impression of Change (PGIC)

The PGIC is a single-item rating of the participant’s impression of change in their condition with treatment on a 7-point scale that ranges from “very much improved” to “very much worse” with “no-change” as the midpoint. The PGIC has frequently been used as an indicator of meaningful change in response to treatments for chronic pain [23]. Consensus guidelines outline the PGIC measure as an important indicator of meaningful change in treatments for chronic pain [24, 25].

##### Patient Global Satisfaction Scale (PGSS)

The PGSS is a single-item rating by participants of their satisfaction with treatment on a 10-point scale that ranges from “very satisfied” to “not at all satisfied”.

##### High-Sensitivity C-Reactive Protein (hs-CRP)

CRP is an acute-phase protein released into the blood by the liver during inflammation and is a sensitive marker of low-grade systemic inflammation. Plasma CRP levels can increase dramatically after severe trauma, bacterial infection, inflammation, surgery, or neoplastic proliferation. Measurement of CRP has been used historically to assess activity of inflammatory disease and to monitor inflammatory processes. The hs-CRP test is a highly sensitive quantification of CRP that can be detected at a lower level than CRP [26].

##### Erythrocyte Sedimentation Rate (ESR)

ESR is a laboratory test for assessing inflammatory or the acute phase response. It is not diagnostic of any particular disease, but when elevated may indicate the presence of inflammation, infection, rheumatologic disease or neoplasm [27].

##### Multi-Systems Adverse Event Monitoring form

Adverse events were tracked and monitored using the Multi-Systems Adverse Event Monitoring form, a standardized, 91-point monitoring form that asks questions pertaining to the following organ systems: eyes/ears/nose/throat, gastrointestinal, neurological/ musculoskeletal, psychological/general, cardiopulmonary, skin, genitourinary, and whole body systems.

### Data security and storage

This study used REDCap—a secure, web-based application that supports electronic data capture for research studies—for data storage and management. Data was exported from REDCap to either Excel or SPSS for analysis. All procedures conducted adhered to the Informed Consent and protocol, as approved by the MetaG SPM IRB.

### Analysis

All 3 time points (Baseline, Week 2 study visit, and Week 4 study visit) for the primary and exploratory outcome measures were initially analyzed using linear mixed modeling, followed by pairwise T-tests between Baseline and Week 2, then Baseline and Week 4, for each outcome measure. For all analyses, statistical significance was set at *p* = 0.05. Where applicable, the raw scores from each outcome measure (primary and exploratory) were translated into T-scores at Baseline, Week 2, and Week 4. These T-scores were then reported as the final score for each patient. Results were reported as “per protocol” analyses, without imputation of missing data, due to the need to determine efficacy.

All but one of the PROMIS-43 data sets, as well as the summary BPI interference score data set, were found to have reasonably normal T-score distributions; non-parametric testing confirmed these results. However, all other questionnaire data sets exhibited non-parametric distributions—this was likely due to the relatively small scales with which these tools are scored. Thus, these data sets were retested using Wilcoxon’s Signed-Rank test or Friedman’s test.

The distribution of hs-CRP showed rightward skew, but this was largely corrected with a log-transformation. All analyses of hs-CRP therefore used T-tests of the log-transformed variable. The distribution of ESR showed a more severe skew that could not be remedied by any standard transformation; therefore, results were verified using the same non-parametric tests listed above.

The PGIC and the PGSS were asked only at one time point (study visit 3); thus, no formal comparisons were performed and only frequency data was reported.

### Sample size and statistical power

This study was powered to detect clinically significant changes in the Physical Function or Fatigue subscales of the PROMIS-43 or in the ACPA QOL Scale. Earlier work on the indicated PROMIS-43 subdomains indicates that minimally significant differences between groups are generally in the range of 4–6 points on a T-scale, corresponding to a standardized effect size of d = 0.4–0.6 [28]. For within-group changes over time, which tend to show larger effects, these estimates should be conservative; and we therefore calculated power to detect an effect of d = 0.5. For the ACPA, less information is available, but we calculated power to find a change of one point; and for an 11-point scale, we reasonably estimated the standard deviation at 2 points. Assuming a correlation of r = 0.5 between pre- and post-treatment measures, this again yielded an effect size of d = 0.5. Finally, using a paired t-test design with a two-sided α = 0.05, we calculated that with 40 participants, we would have 87% power to detect an effect size of d = 0.5 in any of the primary outcome measures. With 20% attrition (32 participants for analysis), we would still retain 78% power. Note that, although the referenced effect sizes are for Physical Function and Fatigue, PROMIS-43 T-scales are scored to have equal means and standard deviations, and we would expect similar power estimates on all subdomains. All power calculations were made using G*Power v.3.1.9.2 [29].

## Results

**Fig. 1:**
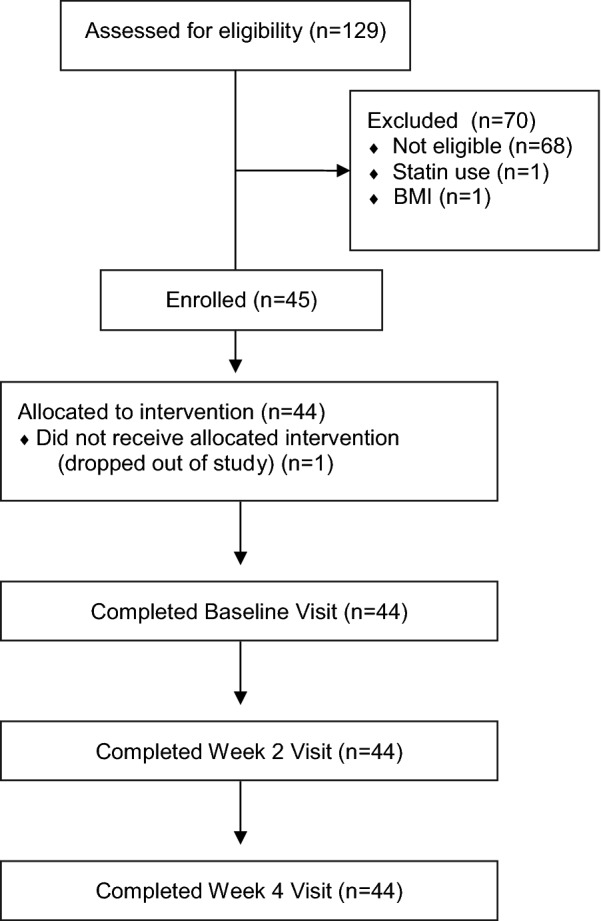
CONSORT diagram of study enrollment and retention

Baseline characteristics are summarized in Table 1. At Baseline, study participants had a mean age of 45.5 years (SD = 13.3), with 31 identifying as female (70.5%), 12 identifying as male (27.3%), and one identifying as transgender (2.3%). Of the 129 individuals screened for inclusion, 45 were found to be eligible and enrolled into the study. Of the 45 enrolled participants, one dropped out of the study, and the remaining 44 participants completed the study (see CONSORT diagram, Fig. 1). Baseline mean primary and exploratory outcome measure results can be found in Table 1.

**Table 1:** Baseline characteristics and instrument scores of study participants

Characteristic (n = 44)	Mean (n%)
Age (years)	45.5 (13.3)
Gender	
Female	31 (70.5)
Male	12 (27.3)
Transgender	1 (2.3)
Currently taking omega-3, fish oil or krill oil	
Yes	14 (31.8)
No	30 (68.2)
Ethnicity	
Hispanic or Latino	3 (6.8)
Not Hispanic or Latino	37 (84.1)
Unknown/not reported	4 (9.1)
Race	
African American	1 (2.3)
Caucasian	34 (77.3)
African American and Caucasian	1 (2.3)
Caucasian and Native American	2 (4.5)
Asian and Pacific Islander	1 (2.3)
Multi-racial (not specified)	2 (4.5)
Unknown/not reported	3 (6.8)

Instrument scores (n = 44)	Mean (SD)
Primary outcome measures	
ACPA QOL scale	6.8 (0.3)
PROMIS-43 QOL subdomains	
Ability to participate in social roles and activities	45.5 (1.3)
Fatigue	54.3 (1.5)
Sleep disturbance	53.8 (1.2)
Exploratory outcome measures	
PHQ-9	7.3 (0.81)
PROMIS-43, depression subdomain	52.2 (1.31)
GAD-7	6.3 (0.66)
PROMIS-43, anxiety subdomain	56.9 (1.36)
BPI, pain intensity items	
Pain now	4.8 (0.28)
Average pain	5.3 (0.18)
Worst pain	6.7 (0.18)
Least pain	3.3 (0.28)
PROMIS-43, pain intensity subdomain	5.7 (0.19)
BPI, pain interference	5.0 (0.36)
PROMIS-43, pain interference subdomain	61.2 (0.95)
PROMIS-43, physical function subdomain	41.3 (1.03)
BPI, pain relief item	3.8 (0.36)
hs-CRP	0.08 (0.19)
ESR	5.18 (0.81)

### Primary outcome results

**Fig. 2:**
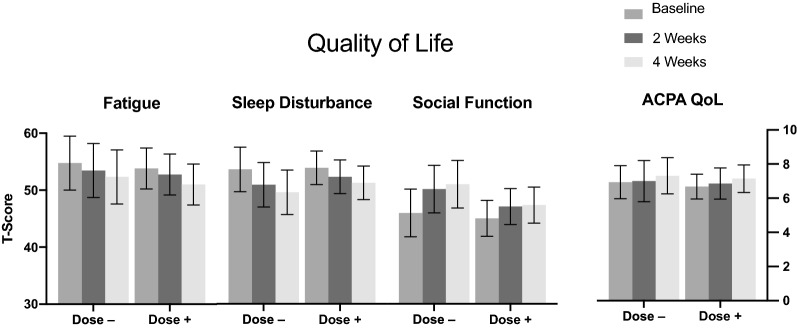
Quality of Life: T-scores (with 95% CIs) for Quality of Life PROMIS-43 subdomains (Fatigue, Sleep, and Social Function); and ACPA Quality of Life scale, by dosing subgroups between Baseline, Week 2, and Week 4 measurements. Dose −: (N = 16) dose decreased at Week 2 study visit; Dose + : (N = 28) dose increased at Week 2 study visit

The T-scores of all 4 quality of life PROMIS-43 subdomains can be found in Fig. 2, which shows summary results by dosing group at the Week 2 visit, i.e. those participants who increased versus those who decreased their dose at Week 2 based on their reported response. Pairwise comparisons for quality of life, as measured by the PROMIS-43 subdomains Fatigue, Sleep Disturbance, and Ability to Participate in Social Roles and Activities (i.e. social function) revealed an overall association between SPM Active**™** use and increased quality of life. Individual pairwise comparisons, presented as the mean difference between Baseline and Week 4, are as follows: a decrease in fatigue of -2.61 [95% Confidence Interval (CI) (− 4.19, − 0.40); *p* < 0.02], a decrease in sleep disturbance of -3.35 [95% CI (− 5.21, − 1.49); *p* < 0.001]; an increase in physical function of 3.40 [95% CI (2.27, 4.52); *p* < 0.001); and an increase in social functioning of 3.70 (95% CI (2.10, 5.30); *p* < 0.001].

Changes between Baseline and Week 4 study visits in the ACPA Quality of Life Scale demonstrated a borderline improvement in quality of life [0.420, 95% CI (− 0.002, 0.841); *p* < 0.051].

### Exploratory outcome results

#### Depression & anxiety

**Fig. 3:**
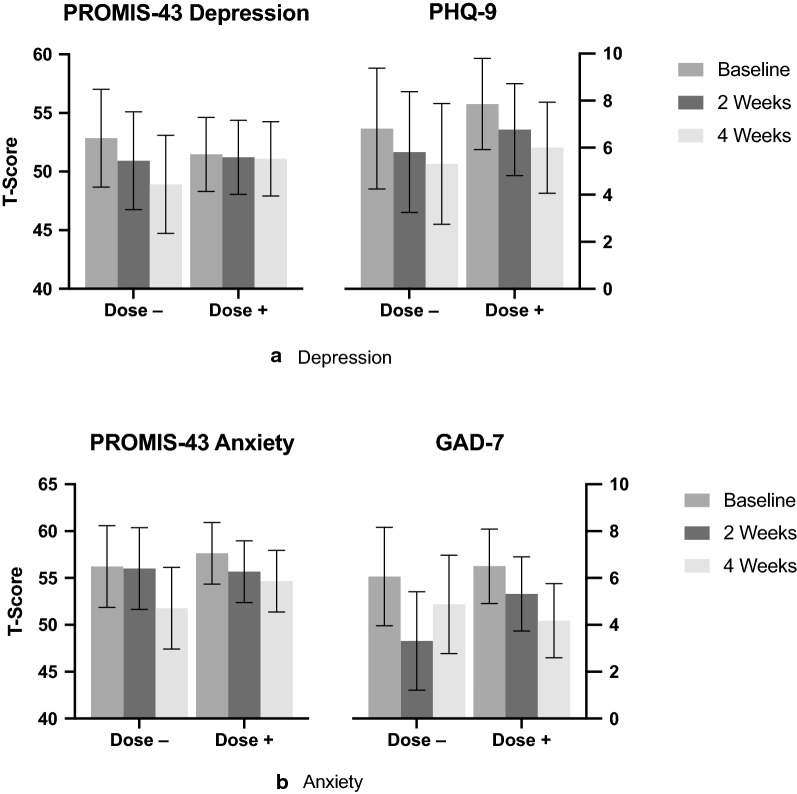
**a** Depression: T-score (with 95% CIs) for PROMIS-43 Depression subdomain, and PHQ-9—comparisons by dosing subgroups between Baseline, Week 2, and Week 4 measurements. Dose −: (N = 16) dose decreased at Week 2 study visit; Dose + : (N = 28) dose increased at Week 2 study visit. **b** Anxiety: T-score (with 95% CIs) for PROMIS-43 Anxiety subdomain, and GAD-7—comparisons by dosing subgroups between Baseline, Week 2, and Week 4 measurements. Dose −: (N = 16) dose decreased at Week 2 study visit; Dose + : (N = 28) dose increased at Week 2 study visit

Pairwise comparison of the mean difference between Baseline and Week 4 in measures of depression, as measured by the PROMIS-43 depression subdomain and the PHQ-9, revealed a decrease in depression of − 2.16 (95% CI (− 3.93, − 0.39); *p* < 0.018) and − 1.68 (95% CI (− 2.82, − 0.54); *p* < 0.004], respectively. Pairwise comparison of the mean difference between Baseline and Week 4 in measures of anxiety, as measured by the PROMIS-43 anxiety subdomain and the GAD-7, revealed a decrease in anxiety of − 3.71 [95% CI (− 5.46, − 1.96]; *p* < 0.0001] and − 1.75 (95% CI (− 2.83, − 0.68); *p* < 0.002], respectively. A comparison of both depression measures and both anxiety measures can be found in Fig. 3a, b below.

#### Changes in pain

**Fig. 4:**
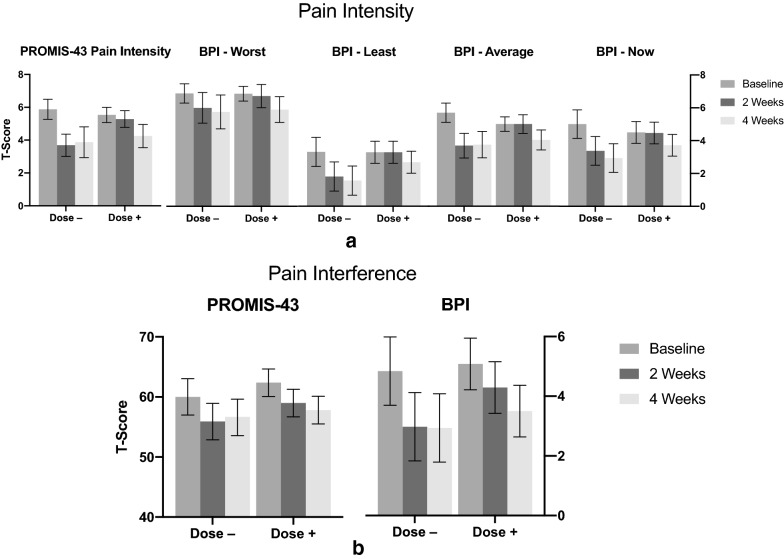
**a **Pain Intensity: T-scores (with 95% CIs) for PROMIS-43 and BPI subdomains of pain intensity—comparison between Baseline, Week 2, and Week 4 measurements. Dose −: (N = 16) dose decreased at Week 2 study visit; Dose + : (N = 28) dose increased at Week 2 study visit. **b** Pain Interference: T-scores (with 95% CIs) for PROMIS-43 and BPI subdomains of pain interference (BPI multiplied by 10 for scaling)—comparison between Baseline, Week 2, and Week 4 measurements. Dose –: (N = 16) dose decreased at Week 2 study visit; Dose + : (N = 28) dose increased at Week 2 study visit

Changes in pain were represented by pain intensity and pain interference, as measured by the PROMIS-43 subdomains of pain intensity and pain interference; and the BPI subdomains of pain interference, worst pain intensity, least pain intensity, pain now, and average pain. Pairwise comparison of the mean difference between Baseline and Week 4 in measures of pain intensity revealed a decrease in PROMIS-43 pain intensity measurements [− 1.64; 95% CI (− 2.17, − 1.12); *p* < 0.0001], as well as in all 4 subdomains of BPI pain intensity: worst [− 1.05; 95% CI (− 1.71, − 0.38]; *p* < 0.002), least (− 1.18; 95% CI (− 1.77, − 0.59); *p* < 0.0001], current [− 1.42; 95% CI (− 2.07, − 0.78]; *p* < 0.0001], and average [− 1.45; 95% CI (− 1.94, − 0.96); *p* < 0.0001]. The T-scores of all five pain intensity measures can be found in Fig. 4a below. The BPI subdomain ‘pain relief’ corroborates the overall association between SPM Active™ supplementation and a decrease in pain intensity [1.07; 95% CI (0.39, 1.74); *p* < 0.002].

Pairwise comparison of the mean difference between Baseline and Week 4 in measures of pain interference, as measured by the PROMIS-43 and BPI subdomains of the same name, revealed a decrease in pain interference of − 3.99 [95% CI (− 5.55, − 2.42); *p* < 0.0001] and − 1.75 [95% CI (− 2.25, − 1.24); *p* < 0.0001], respectively. The T-scores of both pain interference measures can be found in Fig. 4b below.

Pairwise comparison of the mean difference between Baseline and Week 4 in the measure of the PROMIS-43 Physical Function subdomain revealed an increase in physical function of 3.97 [95% CI (2.27, 4.52); *p* < 0.0001].

#### Satisfaction & improvement

**Fig. 5:**
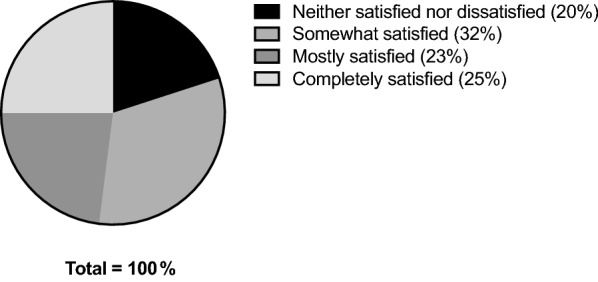
PGSS (*Satisfaction*) at Week 4

**Fig. 6:**
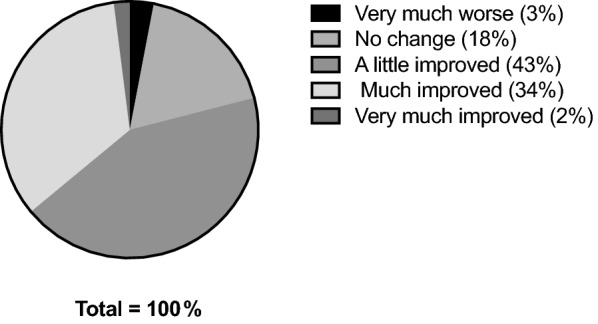
PGIC (*Improvement*) at Week 4

Participant satisfaction with treatment was measured by the PGSS (Fig. 5), and self-assessed impression of change (i.e. improvement; Fig. 6) was measured by the PGIC. Both the PGSS and PGIC were administered only once, at the end of the study, and therefore no formal comparisons could be made.

#### ESR & hs-CRP

Changes in the inflammatory biomarkers ESR and hs-CRP were also analyzed. Results revealed no statistically significant changes in either biomarker, regardless of biomarker status at the start of the study (i.e. elevated or within normal limits). Notably, although laboratory biomarkers did not change significantly during or after supplementation, baseline values were very low for both biomarkers, suggesting the chronic pain reported by participants was independent of inflammation (see Table 1); similar results have been suggested in other trials [30].

## Discussion

The preliminary clinical results reported here support the hypothesis that oral supplementation with a marine lipid fraction enriched and standardized to 17-HDHA and 18-HEPE (as SPM Active**™**) may improve quality of life in an adult population with chronic pain, based on statistically significant changes in the PROMIS-43 subdomains of Fatigue, Sleep Disturbance and Social Functioning. Changes in the ACPA QOL scale also suggested borderline significance, demonstrating consistency in results. In addition, exploratory analyses revealed statistically significant reductions in measures of pain intensity, pain interference, depression, and anxiety, as well as an increase in physical function.

These data are compelling and suggestive of attribution to the intervention for several reasons—first, multiple gold-standard legacy instruments were used within each of the domains measured, and resulted in consistent results. Second, all questionnaire instruments were administered via REDCap, independent of the clinical visits, thus implying that the doctor-patient relationship did not interfere with reporting. Such a mode of questionnaire administration reduces the likelihood of coercion or bias being introduced, thus providing a more robust foundation of credibility. Finally, the intervention showed an association not only with improved pain measures, but with improved depression, anxiety, physical function, and social function measures as well. These results are consistent with the biopsychosocial model of pain, in which physical pain is interrelated with mental and social wellbeing, all 3 of which impact one another and appeared to be modified in our trial [31].

To the best of our knowledge, this is the first report of a clinical trial of an orally administered SPM supplement containing standardized levels of resolvin precursors (17-HDHA and 18-HEPE). Resolvins have been used in several in vitro and in vivo studies to assess their impact on a host of different pathologies, most of them involving some component of inflammation [32–35]. Our findings present new and compelling results that highlight the potential role of SPMs in the treatment not only of the physical component of pain, but of the entire biopsychosocial continuum that contributes to the real life experience of pain. Similar findings have been demonstrated in a mouse fibromyalgia model in which the administration of D-series resolvins reduced pain and depressive symptoms [36]. Additionally, one study explored the direct antidepressant effects of resolvins in mice that were put into a chronic unpredictable stress (CUS) model, which is known to cause depressive-like symptoms in mice. The administration of a single intracerebroventricular injection of D1 and D2 resolvins after having been put through the CUS model resulted not only in significant amelioration of depression-like behaviors within 2 h, this amelioration was still in effect 24 h later [37]. These results suggest that even a single dose of resolvins has the potential to cause rapid and persistent changes in the central nervous system. When paired with the findings from our study—which found a decrease in depression and anxiety—this suggests a possible effect in the central nervous system of humans, or perhaps in gut regulation of neuropeptides. If replicated, these findings could have far-reaching therapeutic implications in the chronic pain population, in which comorbidity with depression is well established [38–41].

Also clinically relevant is the fact that, while research has shown plasma SPM levels to increase with high dose fish oil supplementation in *healthy* individuals, in individuals with pathology present, the rise in 17-HDHA and 18-HEPE in response to high dose fish oil is blunted, suggesting that biosynthetic pathways may be dysregulated [3, 42, 43]. This has been specifically shown in individuals with metabolic syndrome [44]. Similarly, leukocytes isolated from individuals with raised BMI were shown to have an impaired ability to produce SPMs when treated with DHA, and required treatment with 17-HDHA to override this defect [45]. Furthermore, several case–control studies have highlighted a relative deficit of SPMs in patients with a range of conditions, including arthritis, vascular disease, endometriosis, and systemic lupus erythematosus, as well as immediately post-surgery [46–53]. More clinical studies are needed in order to determine what role supplementation with SPMs might play in directing the course of disease in these populations.

There were several limitations to the study, including the short duration of the intervention; the lack of measurement of SPM status at Baseline; and not assessing the bioavailability of the supplement. However, recent use of the emulsion form of the marine lipid oil used in this study as an intervention in a different study showed an acute increase in circulating SPMs in healthy individuals occurring within 2 h of consumption of a single dose, which provides foundational data on which to predict the bioavailability and resulting SPM levels of participants in the current study [16]. The largest limitation of this study was the lack of a control (placebo) group. While clear associations were illuminated in the form of measured changes, the absence of a control group disallows definitive attribution of the measured changes to the intervention. However, despite this limitation, the findings from our trial met all nine Bradford Hill criteria for attribution of causation in a non-randomized controlled trial, suggesting a “true” effect of the intervention [54]. Specifically, the consistent results measured by multiple differing instruments supports causation (criteria of consistency). In addition, several outcomes demonstrated an expected pattern of response based on the dose titration schema used in the trial. Specifically, the PROMIS-43 subdomains of pain interference, physical function, and pain intensity; the BPI subdomains of greatest pain, least pain, average pain, current pain, relief from pain, and pain interference; and the GAD-7 all demonstrated a leveling off in changes in those participants who dose-reduced at the Week 2 visit, and demonstrated improvements in outcomes in those participants who dose-escalated at the Week 2 visit. In short, this is evidence of biological gradient. The study population had been experiencing chronic pain for a minimum of 3 months—a population in which the natural history of the disease does not include spontaneous resolution of symptoms (criteria of specificity) [55]. The magnitude of changes in outcome measures, the identification of consistent results from multiple questionnaires, a dose response consistent with the dose titration protocol, and the specificity of the population all support a true biological effect, and not simply a placebo response.

There were several strengths to the study design. All participants had a certain degree of pain at Baseline in order to be enrolled, thus increasing the likelihood of measuring an effect if present. Data collection was independent of the study encounter, which—as previously discussed—decreases the likelihood of coercion or bias being introduced. Lastly, there is a strong biologic plausibility supporting the intervention as likely to be an effective intervention in a chronic pain population—the therapeutic use of marine oils (and more specifically, the fatty acids they contain) in the treatment of several different pathologies is well-established, as are their impacts on measures of mental and emotional well-being [56–60]. Additionally, the impact of SPMs on analgesic mechanisms in vitro and in reducing pain in several preclinical models in vivo has been previously demonstrated [61, 62].

The clinical significance of these findings has great potential, as the need for efficacious interventions for chronic pain is immense, as is the need for therapies that can affect all aspects of pain (biological, psychological, and social). Such interventions don’t currently exist, and the interventions we do have are—at best—temporarily palliative with high side effect profiles, high incidence of developed resistance, and high likelihood of addiction [2]. To establish a therapeutic agent that is not only effective, but has low to no side effects and is non-addictive, could positively impact the lives of millions of people with chronic pain.

Future research efforts will build on the current study by carefully assessing longer-term safety and efficacy via randomized, placebo-controlled, Phase 1 and Phase 2 trials. In addition, future studies need to explore the bioavailability of marine lipid fractions containing standardized levels of resolvin precursors to help determine a reliable biological signature of effect. Finally, looking at the long-term effects of these pro-resolving supplements in a chronic pain population will be of the utmost importance given the current pain crisis, and the population burden of associated depression, anxiety, and social dysfunction.

## Conclusion

These results report findings from the first clinical trial assessing an orally administered fractionated marine lipid concentration standardized to 17-HDHA and 18-HEPE in a sample of adults with chronic pain, and support the hypotheses that orally administered supplements containing these resolvin precursors may improve the quality of life, reduce pain intensity and interference, and improve mood within 4 weeks of supplementation. Our findings also support the biopsychosocial model of pain and justify the need for well-controlled trials.

## Data Availability

The datasets used and/or analyzed during the current study are available from the corresponding author upon reasonable request.

## Abbreviations

ACPA: American Chronic Pain Association
BMI: Body Mass Index
BPI: Brief Pain Inventory
cGMP: Good Manufacturing Practices
CI: Confidence Interval
CUS: Chronic unpredictable stress
DHA: Docosahexaenoic acid
DSM-IV: Diagnostic and Statistical Manual of Mental Disorders 4
EPA: Eicosapentaenoic acid
ESR: Erythrocyte sedimentation rate
GAD-7: Generalized Anxiety Disorder scale
hs-CRP: High-sensitivity C-reactive protein
IRB: Internal review board
NSAIDs: Non-steroidal anti-inflammatory drugs
NUNM: National University of Natural Medicine
PGIC: Patient Global Impression of Change
PGSS: Patient Global Satisfaction Scale
PHQ-9: Patient Health Questionnaire
PRIME-MD: Primary Care Evaluation of Mental Disorders
PROMIS-43: Patient Reported Outcomes Measurement Information System-43 Profile
QOL: Quality of Life
REDCap: Research Electronic Data Capture
SPM: Specialized pro-resolving mediator
TRP: Transient Receptor Potential
